# Validation of whole room indirect calorimeters: refinement of current methodologies

**DOI:** 10.14814/phy2.13521

**Published:** 2017-11-28

**Authors:** Russell Rising, Thomas Foerster, Avigdor D. Arad, Jeanine Albu, Xavier Pi‐Sunyer

**Affiliations:** ^1^ New York Obesity/Nutrition Research Center Department of Medicine Columbia University New York New York; ^2^ Sable Systems International North Las Vegas, Nevada; ^3^ Department of Endocrinology, Diabetes, and Metabolism Icahn School of Medicine at Mount Sinai New York New York

**Keywords:** Blender, energy, infusion and combustion, propane, validation

## Abstract

Whole room indirect calorimeter (WRIC) validation techniques consist of propane combustion (PC) or infusion of mixed carbon dioxide (CO_2_) and nitrogen (N_2_) by a precision blender (PB). To determine the best method, PC of 6, 10, 22‐h and PB infusions of 6, 10, and 14‐h, were conducted. The 14‐h infusion consisted of two metabolic settings. Energy expenditure (EE; kJ), ventilation (V; liters/min) of oxygen (VO_2_), VCO_2_, and respiratory quotient (VCO_2_/VO_2_) obtained from the WRIC were extrapolated to the respective test durations and compared to similarly calculated values. Moreover, accurate equations (AE) were derived to correct infusions for additional N_2_. As a final evaluation of a PC validated WRIC, weight maintenance (WM), energy balance (EB), respiratory quotient (RQ), and food quotients (FQ) were determined in 22 subjects who had repeat 24‐h EE measurements. Statistical analyses (*P* < 0.05) were conducted (SPSS, version 23). Significant differences in RQ existed between PC and stoichiometry after 6‐h. Errors for the rest of the PC tests ranged from −1.5 ± 2.4 (VCO_2_) to 2.8 ± 4.6% (EE). When compared with the WRIC, all uncorrected metabolic parameters for six and 10‐h PB infusions were significantly different with errors from −12.8 ± 1.6 (VO_2_) to 6.0 ± 2.8% (RQ). The AE reduced the magnitude of errors to −12.4 ± 1.5 (RQ) to 2.2 ± 3.0% (RQ). The PB infusion with two settings showed similar performance. No differences in WM, EB, RQ, or FQ existed in the subjects. In conclusion, 10‐h PC tests are sufficient for validating WRICs.

## Introduction

The Atwater‐Rosa respiration apparatus was the first human 24‐h studies whole room indirect calorimeter (WRIC) in the United States that was completed in 1904 at Wesleyan University, Middletown, CT (Atwater and Benedict 1904). These investigators validated their system by alcohol combustion. They went on to suggest “the factors involved in the combustion or oxidation of food material in the body and the products evolved are the same as in the burning or oxidation of alcohol in a lamp, and the quantities of heat and combustion products, corresponding to a given weight of alcohol, are accurately known” (Atwater and Benedict 1904). Since that time close to 50 modern WRICs have been developed around the world for 24‐h metabolic studies. Many more are planned in the next few years.

Currently, propane combustion (PC) is the most common validation methodology for 24‐h studies WRICs (Ravussin et al. 1986; Shechter et al. 2013, 2014, 2015). Validation in this context refers to combustion of a known substance, such as propane within the WRIC, as a check that all of the individually calibrated components, such as the gas analyzers, flow meters, temperature, and barometric pressure sensors produce correct simulated metabolic results. This is similar in context to that proposed by Atwater and Benedict (1904) when they validated their first WRIC utilizing alcohol combustion. Pure propane body weight maintenance (MW = 44.0 g/mole) combusts, in the presence of an unlimited supply of O_2_ from fresh air, according to the following equation (Withers 2001):

$$C_{3}H_{8}+5O_{2}\to \text{3C}O_{2}+4H_{2}O+\text{49}.\text{87 kJ}/\text{g of heat energy}$$

The consumption of O_2_ and production of CO_2_ equate to an respiratory quotient (RQ) of 0.60. Furthermore, complete oxidation requires an air:fuel ratio of 15.7:1.0 (Hayword 1988). Propane combustion is one of the best simulations of human metabolism under sedentary conditions since it combusts the fuel to CO_2_ and water (Weast 1985; Ravussin et al. 1986). However, one disadvantage of PC is the production of a nonphysiological RQ. The RQ produced by healthy humans, through the metabolism “combustion” of dietary macronutrients, ranges from 0.70 to 1.00 (Atwater and Bryant 1900). Being that the RQ produced by PC is below the physiological norm, it still represents a standard that needs to be achieved by the WRIC to assure its accuracy and precision.

Another validation technique used is mixing and infusion of pure gases such as O_2_, CO_2_ and N_2_ into the WRIC by a precision gas blender (PB) (Charbonnier et al. 1990; Moon et al. 1995; Schoffelen et al. 1997; Tokuyama et al. 2009). The main advantage is that any physiologically relevant metabolic rate and RQ can be created, through various gas mixtures, to simulate that produced by humans consuming diets of various macronutrient compositions. Furthermore, the composition of the infused pure gases can be adjusted or “stepped” to simulate abrupt changes in human metabolic rates and RQs, such as that occurring with exercise. Finally, these infusions are safe since there is no open flame. However, there are several major disadvantages when using a PB for validating a WRIC. For one, complex electronic equipment is needed to regulate the flow of each pure gas to create the mixtures that are infused into the WRIC (Moon et al. 1995). Furthermore, this method is not a true representation of metabolism because, unlike human cellular respiration, there is no “combustion” of a fuel source that produces CO_2_ and water, unless a separate device is incorporated that also injects moisture into the WRIC, along with the infused gases. Even with the additional device to provide moisture, there is still lack of a “combustion” process to produce the respective byproducts. The PB itself only infuses pure dry blended gases into the WRIC (Moon et al. 1995). Moreover, if not accounted for in the calculations incorporated into the PB's software, the infusion of pure N_2_ to create the decrement of O_2_ violates the Haldane transformation (Haldane 1918). Furthermore, if configured similar to the PB used in this study, the purchase cost can be high at approximately US $65,000.00. Finally, the mass flow meters within the PB need periodic calibrations by a laboratory that has National Institute of Standards and Technology certified equipment. Other methods of hard calibrations for the individual mass flow meters within the PB are available such as using oil‐filled wet‐type gas meters. Either way periodic hard calibrations need to be performed to maintain the accuracy of the individual mass flow meters within the PB, leading to additional expense for the necessary equipment and labor to perform the procedures.

The primary objective of this study was to compare simulated metabolic results, utilizing both PC and PB, to their respective calculated values. The study also aimed to determine for each method the shortest duration to validate 24‐h studies WRICs. Finally, we determined WM, energy balance (EB), RQ, and food quotient (FQ) of healthy human subjects from two prior studies (Arad et al. 2015; Hall et al. 2016) that had multiple measurements of 24‐h energy expenditure (EE) under controlled experimental conditions over several weeks in a WRIC that had been previously validated with PC.

## Methods

### Whole room indirect calorimeter

In this study, the 23‐h studies WRIC at the Mount Sinai St. Luke's Hospital in New York City were used for the comparison. This WRIC measures 3.30 × 2.79 × 2.41 m, with an internal volume of 21,399 L, after accounting for that contributed by the furnishings, sink and toilet (Shechter et al. 2013, 2014, 2015; Arad et al. 2015). The respiratory exchange was measured by a Promethion (Model GA3m2/FG250) integrated system (Sable Systems International, Las Vegas NV). This is done by pulling a fixed 80 L/min of fresh air through the WRIC and obtaining a sample on the exhaust side of the system for measurement of O_2_ and CO_2_ concentrations (%). Oxygen and CO_2_ were determined by high‐grade fuel cells and a dual wavelength, nondispersive infrared sensors, respectively. This instrumentation is specified by the manufacturer to be accurate to 1% of reading for mass air flow (liters per minute), and to resolve O_2_ and CO_2_ concentrations to better than 0.001% (Melanson et al. 2010). Water vapor pressure of the sample gas stream is measured directly to 0.001 kilopascals (kPa) by a thin‐film capacitive sensor and used to continuously correct the ventilation rates of O_2_ (VO_2_) and CO_2_ (VCO_2_), along with the total mass air flow. This eliminates the need for desiccants to dry the sample gas stream prior to analysis and the potential errors due to incomplete removal of moisture (Lighton 2008). Moreover, an instantaneous *z*‐transformation mathematical model is applied to the continuous O_2_ and CO_2_ gas concentrations to account for the room's response time to metabolic changes (Bartholomew et al. 1981). Finally, VO_2_ and VCO_2_, along with the RQ (VCO_2_/VO_2_), are then calculated every minute during the metabolic test (Melanson et al. 2010). Thereafter, EE (kcal/day) is calculated with the Weir equation (Weir and De 1949) and expressed as kJ per day by multiplying the result by 4.184.

Prior to each PC or PB infusion the Sable Systems gas analysis instrumentation was calibrated according to the manufacturer's instructions (Melanson et al. 2010).

### Propane gas combustion

**Figure 1:**
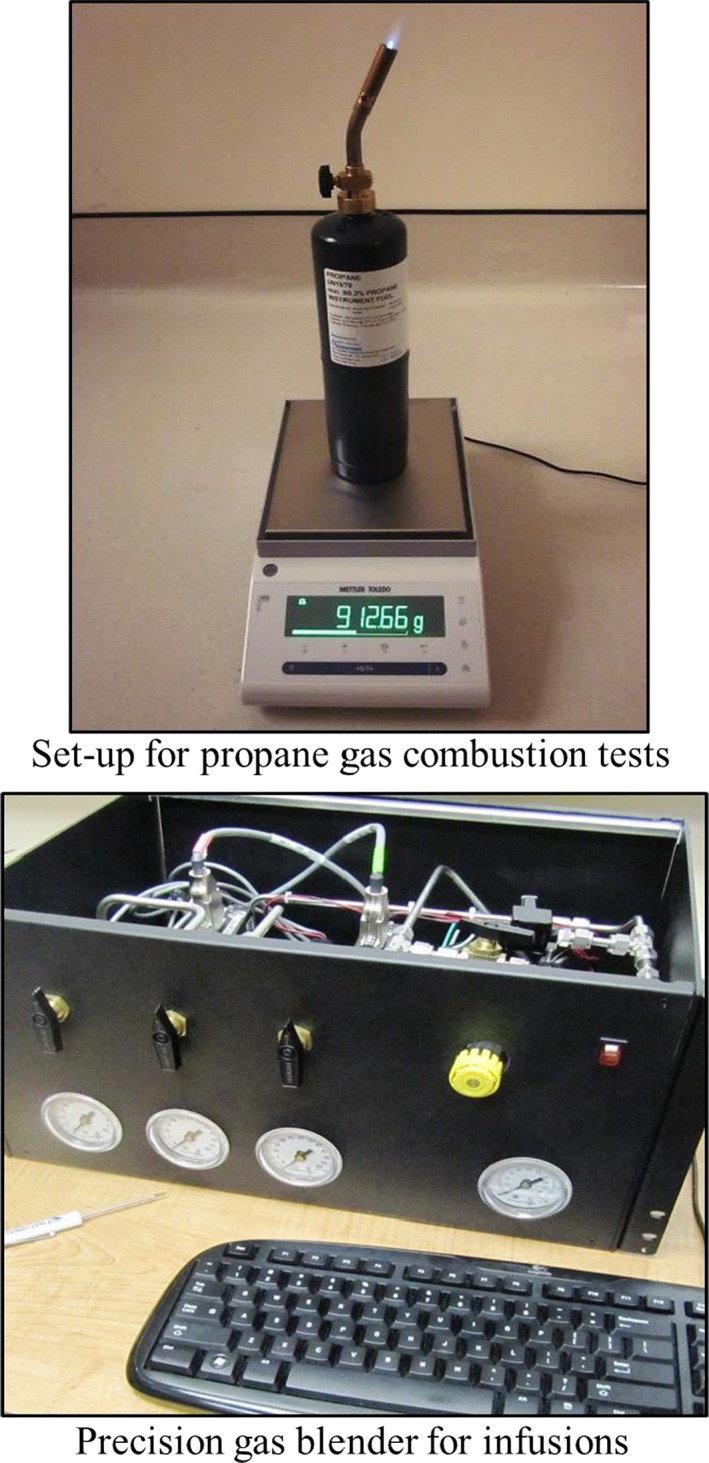
Photos of the set‐up for propane gas combustion (A, top) and that of the precision gas blender (B, bottom).

A total of 27 PC tests were performed in the WRIC, nine each for six, 10 and 22‐h. The first two durations were similar to that of the single‐step PB infusions, whereas the longest one is the most common PC test length currently used to validate 24‐h studies WRIC's (Ravussin et al. 1986; Melanson et al. 2010; Shechter et al. 2013, 2014; Arad et al. 2015; Hall et al. 2016). These PC tests consisted of placing a lecture bottle of instrument grade (99.2% purity) propane (Air Liquide, Houston, TX) and related torch (Model UL2317, Bernzomatic Inc., Chilton, WI) on a calibrated analytical balance (Mettler Toledo Model MS1602S/03, Mettler Toledo LLC, Columbus, OH) as shown in Figure 1A in the center of the WRIC. The stoichiometry (Withers 2001) of the oxidation of propane for EE, VO_2_, VCO_2_, and RQ, were compared to that obtained through actual combustion in the WRIC. The burn rate (g/min) for propane was determined by obtaining the weight of the lecture bottle and torch prior to and after completion of each combustion test. The accuracy of the analytical balance was confirmed prior to the initiation of each PC test by placing calibration weights of the expected ranges on its platform. Every PC test was conducted with the propane lit and the flame set to approximately one inch in height, the WRIC door closed and observed for 5‐min to ensure its integrity prior to starting the test. This procedure is similar to that for human subjects, whereby the metabolic test is started a few minutes after closing the door of the WRIC (Shechter et al. 2013, 2014, 2015; Arad et al. 2015).

### Precision gas blender infusions

For this part of the study a PB (MEI Research Ltd, Edina, MN) was utilized (Figure 1B). The PB utilized in our study was comprised of three mass flow meters (controllers) that are used for calibrated piston prover instruments (Mesa Labs, Butler, NJ). They were certified against National Institute of Standards and Technology precision mass flow meters at the rates used for this study (Table 1). The PB mixed and infused certified pure N_2_ and CO_2_ gases (Air Liquide, Houston, TX) into the WRIC according to the flow protocol shown in Table 1. This flow protocol was designed to simulate single and changing levels, or steps, of sedentary metabolism of a healthy human and depicted as “expected” values in Table 1. Infusions involved connecting tubing to the yellow single gas outlet (Figure 1B) on the front of the PB. Internally, each of the outputs from the individual mass flow meters for CO_2_ and N_2_ were routed to this final gas outlet which was connected to a long piece of tubing that passed through one of the ports of the WRIC. The infused gas was emitted from the end of the tubing that was placed on the floor in the center of the WRIC, similar to placement of the propane gas lecture bottle during PC tests. Similar to PC, the door of the WRIC was closed, the N_2_ and CO_2_ calibration gas tanks turned on and the infusion protocol started by initiating the PBs software (MEI Research LTD, Edina, MN) that was preset to the desired flow protocol (Table 1) and test duration. The number and duration of the pure gas infusions were similar to the PC tests for 6 and 10 h. However, the 14‐h gas infusion was comprised of two “steps” set at 9.5 and 4.5 h, respectively, to simulate a changing human metabolic profile according to “expected” values in Table 1. Finally, as described with the PC tests, the instrumentation of the WRIC was calibrated prior to each PB infusion (Melanson et al. 2010).

**Table 1:** Precision gas blender infusion protocols and related simulated metabolic parameters

Step (no.)	N_2_ flow (L/min)	CO_2_ flow (L/min)	CO_2_ (% of mix)	Expected VO_2_ (L/min)	Expected VCO_2_ (L/min)	Expected RQ (VCO_2_/VO_2_)	Expected energy expenditure (kJ/min)^a^
1	1.300	0.287	18.1	0.330	0.287	0.86	6.82
2	0.928	0.170	15.5	0.230	0.170	0.74	4.56

a *N* = 9 for each step.

### Human subjects

In two prior studies (Arad et al. 2015; Hall et al. 2016) WB and EB (energy intake – expenditure) were determined in human subjects who had at least two repeat metabolic measurements in our WRIC, previously validated by PC. In the first study (Arad et al. 2015), 18 healthy Afro‐American women (20–40 years old, 72.7–109.5 kg) were studied to determine their metabolic flexibility prior to and after a 14‐day exercise intervention, whereas consuming a high fat weight maintenance diet (35% carbohydrate, 50% fat, 15% protein). Initial dietary energy needs, including for exercise, were determined for each subject prior to the initiation of the dietary protocol utilizing a ventilated hood connected to a metabolic cart (Vmax Encore, Carefusion Inc. San Diego, CA). Furthermore, dietary composition was derived utilizing the USDA‐NDSR nutrient data base by a registered dietitian and all meals were provided to the subjects for the entire out‐patient study (Arad et al. 2015). After 9 days on the study diet, initial metabolic measurements (period 1) in the WRIC were performed and dietary energy needs were again adjusted by the registered dietitian prior to the 14‐day exercise intervention. Upon completion of the exercise intervention, metabolic measurements were repeated in the WRIC (period 2). In the second prior study (Hall et al. 2016), WM and EB of four healthy men (36.8 ± 4.5 years and 65 to 125 kg) that resided on the metabolic ward were measured in the WRIC. They each had a total of 16 measurements of 24‐h EE weekly for 2‐months; eight metabolic measurements consuming an isocaloric baseline diet (48.1% carbohydrate, 35.6% fat and 16.1% protein) and eight after being switched to a high‐fat “ketogenic” isocaloric diet (5.9% carbohydrate, 77.3% fat, and 16.9% protein). The first 4 weekly 24‐h EE measurements on each diet were used to determine caloric intake requirements (weeks 1–4), whereas the last four (weeks 5–8) measurements in the WRIC were used for comparison analysis (Hall et al. 2016). Dietary energy and composition were verified with bomb calorimetry and chemical analysis, respectively. Compliance was assured by an onsite registered dietitian through regular meetings with study subjects on the metabolic ward. Finally, their activity levels were continuously monitored to assure compliance to all study protocols.

When subjects are in long‐term EB the FQ, the theoretical RQ produced by the diet when metabolized, should equal the RQ from the WRIC (Flatt 1993). As an additional check of the validity of the human subject data from our WRIC, the FQ was calculated (Toubro et al. 1998) for the respective studies’ (Arad et al. 2015; Hall et al. 2016) dietary compositions for all subjects and compared to the respective RQ's obtained from the WRIC.

The two prior studies cited (Arad et al. 2015; Hall et al. 2016) were approved by the Institutional Review Boards of both St. Luke's‐Roosevelt Hospital and Columbia University, and were conducted in accordance with the Declaration of Helsinki of 1975 as revised in 1983. All participants provided written informed consent before enrollment.

### Calculations

All metabolic data from the WRIC, whether derived from PC, PB, or from the human subjects, were calculated as minute‐by‐minute means using the Promethion instruments Expedata software (Sable Systems International, Las Vegas; version 1.4.15). The PB's software (MEI Research LTD, Edina, MN) was used to calculate the simulated metabolic values for six, 10 and 14‐h for VO_2_, VCO_2_, RQ, and EE. Simulated metabolic values obtained from the WRIC for all the validations (PC and PB) were then multiplied by the total test duration (minutes). The human subject data were calculated in a similar manner but extrapolated to 24‐h by multiplying the minute‐by‐minute mean by 1440 (minutes in 24‐h). However, the software in our particular PB did not account for the changes in the Haldane transformation (Haldane 1918) due to the infusion of additional N_2_. For the 14‐h test duration, this equaled an additional 1036 L of N_2_ infused, representing 4.6% of the total volume of the WRIC. To correct for the violation of the Haldane transformation (Haldane 1918), accurate equations (AE) were derived and applied to the data produced by the PB as shown in Table 2. This derivation is similar to a prior study where additional N_2_ infusion was accounted for during the validation of the Deltatrac Metabolic Monitor for use in hospitalized patients connected to ventilators (Takala et al. 1989).

**Table 2:** Derivation of accurate equations (AE) to account for violation of the Haldane transformation (Haldane 1918) by the PB software due to additionally infused nitrogen

Equations
The Haldane transformation assumes that N_2_ is physiologically inert (Haldane 1918). Therefore, the volume (V) of inspired (i) and expired (e) fractions (F) of N_2_ must be equal according to the following formulas: (1) $\text{Vi}\times \text{Fi}N_{2}=\text{Ve}\times \text{Fe}N_{2}$ (2) $\text{Vi}=\left(\text{Ve}\times \text{Fe}N_{2}\right)/\text{Fi}N_{2}$ (3) $\text{Vi}=\text{Ve}\times \left(\text{Fe}N_{2}/\text{Fi}N_{2}\right)$
According to Dalton's law (for dry air) for the incurrent and excurrent flows: (4a) $\text{Fi}N_{2}=1-\text{Fi}O_{2}-\text{FiC}O_{2}$ (4b) $\text{Fe}N_{2}=1-\text{Fe}O_{2}-\text{FeC}O_{2}$
Substituting in (equation 3), therefore: (5) $\text{Vi}=\text{Ve}\times \left(1-\text{Fe}O_{2}-\text{FeC}O_{2}\right)/\left(1-\text{Fi}O_{2}-\text{FiC}O_{2}\right)$
The balance equation for O_2_ flow: (6a) $VO_{2}=\text{Vi}\times \text{Fi}O_{2}-\text{Ve}\times \text{Fe}O_{2}$ (6b) $VO_{2}=\text{Ve}\times \left[\left(1-\text{Fe}O_{2}-\text{FeC}O_{2}\right)/\left(1-\text{Fi}O_{2}-\text{FiC}O_{2}\right)\right]\times \text{Fi}O_{2}-\text{Ve}\times \text{Fe}O_{2}$ Then: (6c) $VO_{2}=\text{Ve}\times \left[\left(1-\text{Fe}O_{2}-\text{FeC}O_{2}\right)\times \text{Fi}O_{2}-\left(1-\text{Fi}O_{2}-\text{FiC}O_{2}\right)\times \text{Fe}O_{2}\right]/\left(1-\text{Fi}O_{2}-\text{FiC}O_{2}\right)$ Finally: (6d) $VO_{2}=\text{Ve}\times \left[\left(1-\text{FeC}O_{2}\right)\times \text{Fi}O_{2}-\left(1-\text{Fi}O_{2}\right)\times \text{Fe}O_{2}\right]/\left(1-\text{Fi}O_{2}-\text{FiC}O_{2}\right)$
The balance equation for CO_2_ flow: (7a) $\text{VC}O_{2}=\text{Ve}\times \text{FeC}O_{2}-\text{Vi}\times \text{FiC}O_{2}$ (7b) $\text{VC}O_{2}=\text{Ve}\times \text{FeC}O_{2}-\text{Ve}\times \left(1-\text{Fe}O_{2}-\text{FeC}O_{2}\right)/\left(1-\text{Fi}O_{2}-\text{FiC}O_{2}\right)\times \text{FiC}O_{2}$ Then: (7c) $\text{VC}O_{2}=\text{Ve}\times \left[\left(1-\text{Fi}O_{2}-\text{FiC}O_{2}\right)\times \text{FeC}O_{2}-\left(1-\text{Fe}O_{2}-\text{FeC}O_{2}\right)\times \text{FiC}O_{2}\right]/\left(1-\text{Fi}O_{2}-\text{FiC}O_{2}\right)$ Finally: (7d) $\text{VC}O_{2}=\text{Ve}\times \left[\left(1-\text{Fi}O_{2}\right)\times \text{FeC}O_{2}-\left(1-\text{Fe}O_{2}\right)\times \text{FiC}O_{2}\right]/\left(1-\text{Fi}O_{2}-\text{FiC}O_{2}\right)$
The calculations for equation numbers 6 and 7 represent the VO_2_ and VCO_2_ readings of the Promethion system after data processing (from here on denoted as VO_2_* and ${\text{VCO}}_{2}^{}*$). In the case of no N_2_ exchange within the WRIC (e.g., normal subject or propane burn), VO_2_ = VO_2_*; the reading of the system matches the true oxygen exchange of the subject. In the case of an N_2_ injection the identity between instrument reading and true oxygen exchange does not hold any longer. In fact, this mismatch is the basis of the injection method. This is because the additional N_2_ and CO_2_ infused is a substitute for the incurrent air flow which results in less flow of actual O_2_ and CO_2_ suggesting “apparent” consumption. To derive a prediction of the instrument reading during active injection of N_2_, we reformulated the N_2_ balance in the WRIC explicitly. As before, the indices “e” and “i” equal excurrent and incurrent WRIC air flow, respectively, index “k” refers to the infused flow properties. Augmenting the Haldane transformation (Haldane 1918) by the injected N_2_ flow: (8) $$\text{Vk}\times \text{Fk}N_{2}=\left(\text{Ve}\times \text{Fe}N_{2}\right)-\left(\text{Vi}\times \text{Fi}N_{2}\right)$$
Solving for Vi: (9) $\text{Vi}=\text{Ve}\times \left(\text{Fe}N_{2}/\text{Fi}N_{2}\right)-\text{Vk}\times \left(\text{Fk}N_{2}/\text{Fi}N_{2}\right)$
If the infused gases are dry and O_2_ free (i.e., FkH_2_O = 0, FkO_2_ = 0), Dalton's law for the injected flow results in: (10) $\text{Fk}N_{2}=1-\text{FkC}O_{2}$
Since in that case also no actual O_2_ exchange occurs (VO_2_ = 0), eq (6) simplifies to: (11a) $0=\text{Vi}\times \text{Fi}O_{2}-\text{Ve}\times \text{Fe}O_{2}$ (11b) $\text{Vk}\times \text{FkC}O_{2}=\text{Ve}\times \text{FeC}O_{2}-\text{Vi}\times \text{FiC}O_{2}$
Substituting (equation 8) and (equation 9) into (equation 10) yields: (12a) $0=\left\{\text{Ve}\times \left[\text{Fi}O_{2}\times \left(1-\text{Fe}O_{2}-\text{FeC}O_{2}\right)-\text{Fe}O_{2}\times \left(1-\text{Fi}O_{2}-\text{FiC}O_{2}\right)\right]/\left(1-\text{Fi}O_{2}-\text{FiC}O_{2}\right)\right\}-\text{Vk}\times \left[\text{Fi}O_{2}\times \left(1-\text{FkC}O_{2}\right)\right]/\left(1-\text{Fi}O_{2}-\text{FiC}O_{2}\right)$ (12b) $\text{Vk}\times \text{FkC}O_{2}=\text{Ve}\times \text{FeC}O_{2}-\left[\text{Ve}\times \left(\text{Fe}N_{2}/\text{Fi}N_{2}\right)-\text{Vk}\times \left(\text{Fk}N_{2}/\text{Fi}N_{2}\right)\right]\times \text{FiC}O_{2}$ (12c) $\text{Vk}\times \text{FkC}O_{2}=\left\{\text{Ve}\times \text{FeC}O_{2}-\text{Ve}\times \left(\text{Fe}N_{2}/\text{Fi}N_{2}\right)\times \text{FiC}O_{2}\right\}+\text{Vk}\times \left(\text{Fk}N_{2}/\text{Fi}N_{2}\right)\times \text{FiC}O_{2}$
Note that the first summands on the right‐hand sides are identical to equations (7a and b) above, which give the system's reading of ${\text{VO}}_{2}^{*}$ and ${\text{VCO}}_{2}^{*}$ (marked by {}). Thus, the predicted readings for a given injected flow Vk, FkN_2_, FkCO_2_ are as follows: (13a) $${\text{VO}}_{2}^{*}\;\text{predicted}=Vk^{}\times \text{Fi}O_{2}\times \left(1_{}-\text{FkC}O_{2}\right)/\left(1-\text{Fi}O_{2}-\text{FiC}O_{2}\right)$$ (13b) $${\text{VCO}}_{2}^{*}\;\text{predicted}=\text{Vk}\times \left(\text{FkC}O_{2}+\text{FkC}O_{2}\times \text{FiC}O_{2}-\text{FiC}O_{2}\right)/\left(1-\text{Fi}O_{2}-\text{FiC}O_{2}\right)$$
These predictions can then be used to define a “recovery” (RO_2_; RCO_2_) as: (14a) $RO_{2}={\text{VO}}_{2}^{*}\;\text{observed}/{\text{VO}}_{2}^{*}\;\text{predicted}$ (14b) $RO_{2}={\text{VO}}_{2}^{*}\;\text{observed}\times \left(1-\text{Fi}O_{2}-\text{FiC}O_{2}\right)/\left[\text{Vk}\times \text{Fi}O_{2}\times \left(1-\text{FkC}O_{2}\right)\right]$ (14c) $\text{RC}O_{2}={\text{VCO}}_{2}^{*}\;\text{observed}/\text{VC}O_{2}*\;\text{predicted}$ (14d) $\text{RC}O_{2}={\text{VCO}}_{2}^{*}\;\text{observed}\times \left(1-\text{Fi}O_{2}-\text{FiC}O_{2}\right)/\left[\text{Vk}\times \left(\text{FkC}O_{2}+\text{FkC}O_{2}\times \text{FiC}O_{2}-\text{FiC}O_{2}\right)\right]$

### Statistics

Statistical analyses were performed using SPSS (version 23, Chicago, IL). Independent t‐tests (*P* < 0.05) were utilized for the comparisons between total VO_2_, VCO_2_, RQ, and EE obtained from PC to that of stoichiometry for test durations of six, 10 and 22‐h. One‐way ANOVA, with post hoc Bonferroni correction for multiple comparisons (*P* < 0.05), was used to determine the differences between total VO_2_, VCO_2_, RQ, and EE from the WRIC to that calculated by the PB's software, uncorrected for the additional N_2_ infusion, and with utilization of the AE (Table 2) to account for the violation of the Haldane transformation (Haldane 1918). A similar analysis utilizing independent *t*‐tests (*P* < 0.05) was conducted to determine any differences in WM, EB, RQ, and the FQ in the human subjects (Arad et al. 2015; Hall et al. 2016).

## Results

### Propane gas combustion

During a 6‐h test duration (Table 3), there were no differences (*P* = NS) between PC and stoichiometry for VO_2_, VCO_2_, and EE. However, a significant difference (*P* < 0.05) did exist for the RQ between PC and stoichiometry (Table 3). The percentage difference between PC and stoichiometry ranged from −2.8 ± 2.4 for the RQ, to 2.8 ± 4.6% for EE (Table 3).

**Table 3:** Comparison of propane combustion against stoichiometry during three different test durations

	Comparison		
Parameter	WRIC	Stoichiometry	Delta % (WRIC‐PC^a^)
6‐h^b^ (burn rate = 0.1388 ± 0.0339 g/min)			
VO_2_ (liters)	24.8 ± 29.6	27.1 ± 31.0	1.6 ± 5.3
VCO_2_ (liters)	77.4 ± 19.1	76.3 ± 18.6	−1.5 ± 2.4
RQ (VCO_2_/VO_2_)	0.62 ± 0.01	0.60 ± 0.00^c^	−2.8 ± 2.4
Energy expenditure (EE) (kJ)	2416.8 ± 575.3	2490.9 ± 606.6	2.8 ± 4.6
10‐h^b^ (burn rate = 0.1780 ± 0.0215 g/min)			
VO_2_ (liters)	273.2 ± 32.5	271.9 ± 32.8	−0.5 ± 1.6
VCO_2_ (liters)	164.1 ± 19.8	163.1 ± 19.7	−0.6 ± 0.9
RQ (VCO_2_/VO_2_)	0.60 ± 0.01	0.60 ± 0.00	0.5 ± 1.9
EE (kJ)	5263.6 ± 627.5	5326.5 ± 643.2	1.2 ± 1.5
22‐h^b^ (burn rate = 0.1419 ± 0.0221 g/min)			
VO_2_ (liters)	484.9 ± 78.5	476.7 ± 74.1	−1.6 ± 1.6
VCO_2_ (liters)	289.5 ± 43.5	286.0 ± 44.5	−1.3 ± 0.8
RQ (VCO_2_/VO_2_)	0.60 ± 0.01	0.60 ± 0.00	0.1 ± 1.7
EE (kJ)	9335.6 ± 1495.0	9340.9 ± 1451.8	0.1 ± 1.4

a Propane combustion.

b *N* = 9.

c Stoichiometry significantly (*P* < 0.05) different from WRIC by independent *t*‐test.

When the test duration was lengthened to 10‐h, no differences (*P* = NS) between PC and stoichiometry were detected for VO_2_,VCO_2_, RQ, and EE (Table 3). This resulted in all of the percent differences between PC and stoichiometry ranging from −0.5 ± 1.6 for the VO_2_, to 1.2 ± 1.5% for EE (Table 3). Finally, when the test duration was lengthened to 22‐h, no differences (*P* = NS) were found for VO_2_, VCO_2_, RQ, and EE (Table 3). Moreover, the percent differences ranged from −1.3 ± 0.8 for the VCO_2_, to 0.1 ± 1.4% for EE (Table 3).

### Precision gas blender with a single step

During a 6‐h test duration (Table 4), VO_2_,VCO_2_, RQ, and EE, calculated with the PBs software, were all significantly different (*P* < 0.05) from those obtained from the WRIC. The differences between the PB and the WRIC in the aforementioned metabolic parameters remained statistically significant (*P* < 0.05) even after the application of the AE, albeit slightly lower (Table 4). The differences between all the measured uncorrected metabolic parameters between the WRIC and the PB ranged from −12.8 ± 1.6 for VO_2,_ to −5.0 ± 1.5% for the RQ (Table 4). The use of the AE did not reduce these percentages in comparison to those of the WRIC (Table 4). Using a 10‐h test duration did not reduce the differences (*P* < 0.05) between VO_2_, RQ, and EE between the WRIC and the PB. However, there were no differences detected with regard to the VCO_2_ (Table 4). This is expected, since the Haldane correction for nitrogen dilution is negligible in the case of CO_2_ because of its far smaller initial concentration, as compared to oxygen. Furthermore, the use of the AE at this test duration did not eliminate these differences (*P* < 0.05) except for VO_2_ (Table 4). However, the longer test duration of 10‐h, without the use of the new equations, did slightly reduce the range of the percent differences between the PB and WRIC (Table 4) from −4.3 ± 1.4 for VO_2_, to 6.0 ± 2.8% for the RQ. Using the AE to account for the additional infused N_2_ reduced the range of these percentages slightly further to −0.9 ± 1.1 for EE and to 2.2 ± 3.0% for the RQ (Table 4).

**Table 4:** Comparison of the whole room indirect calorimeter against the precision gas blender and the accurate equations (AE) during six and 10‐h test durations

	Comparison				
Parameter	WRIC	PB^a^	AE^b^	Delta % (WRIC‐PB)	Delta % (WRIC‐AE)
6‐h^c^					
VO_2_ (liters)	93.4 ± 1.3	82.8 ± 0.0^d^	88.6 ± 0.0^d^	−12.8 ± 1.6	−5.4 ± 1.4
VCO_2_ (liters)	66.0 ± 1.0	61.1 ± 0.0^d^	61.2 ± 0.0^d^	−7.9 ± 1.7	−7.8 ± 1.6
Respiratory quotient (RQ) (VCO_2_/VO_2_)	0.78 ± 0.01	0.74 ± 0.00^d^	0.69 ± 0.0^d^	−5.0 ± 1.5	−12.4 ± 1.5
Energy expenditure (EE) (kJ)	1845.0 ± 24.9	1644.4 ± 2.5^d^	1747.2 ± 0.0^d^	−12.2 ± 1.6	−5.6 ± 1.4
10‐h^c^					
VO_2_ (liters)	208.0 ± 2.7	199.4 ± 0.1^d^	206.4 ± 0.0	−4.3 ± 1.4	−0.8 ± 1.3
VCO_2_ (liters)	172.9 ± 1.7	172.2 ± 0.0	171.6 ± 0.0^d^	−0.4 ± 1.0	−0.7 ± 1.0
RQ (VCO_2_/VO_2_)	0.81 ± 0.02	0.86 ± 0.00^d^	0.83 ± 0.00^d^	6.0 ± 2.8	2.2 ± 3.0
EE (kJ)	4230.2 ± 47.8	4086.9 ± 5.0^d^	4192.4 ± 0.0^d^	−3.5 ± 1.3	−0.9 ± 1.1

a Precision gas blender (PB) total extrapolated data based on the “expected” values for the test duration without correction for violation of the Haldane transformation (Haldane 1918).

b AE utilized to recalculate the “expected” values to correct for the Haldane transformation (Haldane 1918) due to additional infused nitrogen.

c *N* = 9 single “step” PB infusions.

d Significantly (*P* < 0.05) different from WRIC by one‐way ANOVA with Bonferroni post hoc.

### Precision gas blender with two steps

For this part of the study the PB mass flow rates were set to the first “step” for nine‐ and‐a‐half hours to produce the metabolic parameters shown in Table 5. Thereafter, the flow settings were changed to the next “step”, as shown in Table 1, for an additional four‐and‐a half hours to produce a new set of metabolic parameters (Table 5). During the first nine‐and‐a‐half‐hours there were significant differences (*P* < 0.05) between the PB and the WRIC for uncorrected VO_2_, RQ, and EE and while that for the VCO_2_ was similar (Table 5). However, differences in VCO_2_, RQ, and EE still existed (*P* < 0.05) with the use of the AE. The only improvement with the AE was in the VO_2,_ with no differences detected (Table 5). The percent differences in the first nine‐and‐a‐half‐hour “step” ranged from −3.5 ± 1.4 for the EE to 6.2 ± 3.0% for the RQ. The AE reduced these errors to – 0.9 ± 1.3 for the EE to 2.5 ± 3.2% for the RQ (Table 5).

**Table 5:** Comparison of the whole room indirect calorimeter against the precision gas blender and the accurate equations (AE) during two different metabolic (step) settings

	Comparison				
Parameter	WRIC	PB^a^	AE^b^	Delta % (WRIC‐PB)	Delta % (WRIC‐AE)
First 9.5‐h step^c^					
VO_2_ (liters)	198.2 ± 2.3	189.4 ± 0.1^d^	196.1 ± 0.0^d^	−4.6 ± 1.2	−1.1 ± 1.2
VCO_2_ (liters)	164.5 ± 1.7	163.6 ± 0.0	163.0 ± 0.0^d^	−0.5 ± 1.0	−0.9 ± 1.0
Respiratory quotient (RQ) (VCO_2_/VO_2_)	0.82 ± 0.02	0.86 ± 0.00^d^	0.83 ± 0.00	5.2 ± 2.1	1.4 ± 2.4
Energy expenditure (EE) (kJ)	4028.5 ± 39.6	3882.4 ± 4.6^d^	3982.7 ± 0.0^d^	−3.8 ± 1.1	−1.1 ± 1.0
Second preceding 4.5‐h step					
VO_2_ (liters)	69.7 ± 0.9	62.1 ± 0.0^d^	66.4 ± 0.0^d^	−12.3 ± 1.4	−4.9 ± 1.3
VCO_2_ (liters)	49.1 ± 0.9	45.8 ± 0.0^d^	45.9 ± 0.0^d^	−7.1 ± 1.9	−7.0 ± 1.9
RQ (VCO_2_/VO_2_)	0.78 ± 0.01	0.74 ± 0.00^d^	0.69 ± 0.00^d^	−6.2 ± 1.5	−13.7 ± 1.6
EE (kJ)	1376.5 ± 16.5	1233.3 ± 1.9^d^	1310.4 ± 0.0^d^	−11.6 ± 1.4	−5.0 ± 1.3
Combined test duration of 14‐h (steps 1 and 2)					
VO_2_ (liters)	264.7 ± 2.6	248.4 ± 0.1^d^	259.6 ± 0.0^d^	−6.6 ± 1.1	−2.0 ± 1.0
VCO_2_ (liters)	210.4 ± 2.0	205.9 ± 0.0^d^	205.4 ± 0.0^d^	−2.2 ± 1.0	−2.4 ± 1.0
RQ (VCO_2_/VO_2_)	0.80 ± 002	0.82 ± 0.00^d^	0.78 ± 0.00^d^	2.3 ± 2.4	−2.5 ± 2.6
EE (kJ)	5339.1 ± 44.7	5048.6 ± 2.8^d^	5229.2 ± 0.0^d^	−5.8 ± 0.9	−2.1 ± 0.9

a Precision gas blender (PB) total extrapolated data based on the “expected” values for the test duration without correction for violation of the Haldane transformation (Haldane 1918).

b AE utilized to recalculate the “expected” values to correct for the Haldane transformation (Haldane 1918) due to additional infused nitrogen gas.

c *N* = 9 two‐step PB infusions of 14‐h.

d Significantly different (*P* < 0.05) from WRIC by one‐way ANOVA with Bonferroni post hoc.

When the PB was switched to the new flow settings for N_2_ and CO_2_ for the second “step”, there were still significant differences (*P* < 0.05) in uncorrected VO_2_, VCO_2_, RQ, and EE (Table 5). The percent differences between the PB and the WRIC were higher for the second four‐and‐a‐half‐hour “step” ranging from −7.2 ± 1.8 for VCO_2_ to −11.7 ± 1.1% for EE (Table 5). The use of the AE did not eliminate any of the differences (*P* < 0.05) for VO_2_, VCO_2_, RQ, and EE for the second “step” (Table 5). Moreover, the percent differences in the second “step” between the PB and the WRIC, corrected with the AE, ranged from −13.2 ± 1.6 for the RQ to −5.1 ± 1.1% for the VO_2_ (Table 5). When both the first and second step duration results were combined to 14‐h, significant differences (*P* < 0.05) still existed for uncorrected VO_2_, VCO_2_, RQ, and EE (Table 5). The percentage difference between the PB and the WRIC across the total 14‐h test duration ranged from −6.6 ± 1.1 for VO_2_, to 2.3 ± 2.4% for the RQ (Table 5). The use of the AE to correct the data from the PB improved these percentages slightly, from −2.5 ± 2.6 for the RQ, to −2.0 ± 1.0% for VO_2_ (Table 5).

### Human subjects

Utilizing PC to validate the WRIC, we found no differences in WM or EB in the 18 women that had repeat measurements of 24‐h EE prior to and after a 14‐day exercise intervention (Table 6). However, EB for the 18 subjects ranged from −3537.2 to 2189.1 kJ/day. Furthermore, no differences existed between the FQ and RQ (Table 6). Similarly, for the four men that had repeat measurements of 24‐h EE over the last month of a 2 month in‐patient study (Table 7), no differences existed in WM and EB during the last 4 weeks on each dietary protocol (Table 7). Energy balance ranged from −512.1 to 988.3 and −594.1 to 988.3 kJ/day for those subjects being fed the standard American and ketogenic diets, respectively. Finally, the FQ was nearly equal to the RQ during the last 4 weeks on each diet (Table 7).

**Table 6:** Body weight maintenance and EB of 18 Afro‐American women fed a high fat diet from a prior study pre and post 14‐weeks of an exercise intervention

Parameter	Period 1	Period 2
Body weight (kg)	86.3 ± 11.2	85.6 ± 11.6
Body mass index (kg/height^2^)	28.7 ± 5.3	31.2 ± 3.2
Energy intake (kJ/d)	8677.8 ± 1331.8	9014.9 ± 1251.7
Energy expenditure (EE) (kJ/d)	8539.8 ± 1023.8	9005.2 ± 1521.9
Energy balance (EB) (kJ/d)	138.1 ± 877.1	9.7 ± 1263.8
Respiratory quotient (VCO_2_/VO_2_)	0.83 ± 0.03	0.82 ± 0.04
Food quotient	0.82 ± 0.00	0.82 ± 0.00

**Table 7:** Body weight maintenance and energy balance of four in‐patient subjects fed isocaloric baseline and “ketogenic” diets

	Comparison			
Parameter	Week 5	Week 6	Week 7	Week 8
Baseline diet (48.1% CHO^a^, 35.6% FAT and 16.1% PRO^b^)^c^				
Body weight (kg)	92.0 ± 25.3	92.1 ± 25.9	91.9 ± 25.1	91.8 ± 25.5
Body mass index (kg/height^b^)	30.0 ± 4.3	30.0 ± 4.4	29.9 ± 4.2	29.9 ± 4.3
Energy intake (kJ/d)	11296.8 ± 0.0	17363.6 ± 0.0	12552.0 ± 0.0	11715.2 ± 0.0
Energy expenditure (EE) (kJ/d)	11552.9 ± 186.5	16905.4 ± 601.8	12258.5 ± 242.4	11721.9 ± 322.1
Energy balance (EB) (kJ/d)	−256.1 ± 186.5	458.1 ± 601.7	293.5 ± 242.4	−6.6 ± 322.1
Respiratory quotient (RQ) (VCO_2_/VO_2_)	0.80 ± 0.03	0.80 ± 0.03	0.81 ± 0.03	0.80 ± 0.04
Food quotient	0.85 ± 0.00	0.85 ± 0.00	0.85 ± 0.00	0.85 ± 0.00
High fat “ketogenic” diet (5.9% CHO, 77.3% FAT and 16.9% PRO)^c^				
Body weight (kg)	90.6 ± 24.3	90.5 ± 24.9	90.9 ± 24.8	90.5 ± 25.2
Body mass index (kg/height^b^)	29.6 ± 4.0	29.5 ± 4.2	29.6 ± 4.2	29.5 ± 4.3
Energy intake (kJ/d)	11296.8 ± 0.0	17363.6 ± 0.0	12552.0 ± 0.0	11715.2 ± 0.0
EE (kJ/d)	11769.2 ± 177.8	16325.6 ± 343.3	11921.1 ± 342.0	11849.2 ± 232.5
EE balance (kJ/d)	−472.4 ± 177.8	1038.1 ± 343.3	630.9 ± 342.0	−134.0 ± 232.5
RQ (VCO_2_/VO_2_)	0.70 ± 0.04	0.71 ± 0.04	0.71 ± 0.02	0.71 ± 0.03
Food quotient	0.74 ± 0.00	0.74 ± 0.00	0.74 ± 0.00	0.74 ± 0.00

a Carbohydrate.

b Protein.

c *N* = 4 subjects measured repeatedly for 16 weeks.

## Discussion

The purpose of this study was to evaluate two methods used for validating WRICs. For this analysis, both PC and PB infusion simulated metabolic parameters, as calculated from source reactants (propane or infused mixture of CO_2_ and N_2_), were compared to that obtained from the WRIC. The results showed that PC is one of the best validation methods for simulating a sedentary human subject's 24‐h metabolism. We also found that a 10‐h PC test is sufficient to provide an accurate validation of the WRIC system utilized for 24‐h human metabolic studies. Using the PB for the same validation tests produced similar results, but only after applying the AE which accounted for violation of the Haldane transformation (Haldane 1918) due to the additionally infused nitrogen. When the PB was “stepped” in order to create a different simulated sedentary metabolic rate, the magnitude of errors was increased during the second metabolic setting. The use of the AE greatly reduced the errors associated with the PB's simulated metabolic results, but not quite to those found for PC tests of up to 22‐h. The variability in repeated PC tests for both 10 and 22‐h showed that all of the simulated metabolic parameters from the WRIC were consistently less than 1.6% of that of stoichiometry. Furthermore, the small standard deviations for these PC tests suggests that the WRIC is a precise, as well as an accurate system for measuring human metabolism. Finally, some of the individual PC test results from our WRIC have shown VO_2_ and VCO_2_ to be within 1% of that of stoichiometry.

The goal of any validation technique is to simulate, as closely as possible, the actual metabolism of a human subject. Even the investigators who built the Atwater‐Rosa respiration apparatus (Atwater and Benedict 1904) realized this when they utilized alcohol combustion to validate their new WRIC. In our study, violating the Haldane transformation (Haldane 1918) during calculation of simulated metabolic parameters, caused by the PB's software, led to our WRIC overestimating EE and the rates for both VO_2_ and VCO_2_, along with variable effects on the RQ. Moreover, if our PB were used as the standard of comparison for human subjects, this would have led to downward adjustments of all the metabolic parameters of up to four percent. This could contribute to consistent underfeeding of energy and thus lead to gradual weight loss in the human subjects during long duration metabolic studies. The best results obtained from the PB, after applying the AE to correct for violation of the Haldane transformation (Haldane 1918), were during the 10‐h infusion tests. The variability for these corrected 10‐h infusions was similar to that for PC tests of the same duration. Moreover, all simulated metabolic parameters, except the RQ, obtained from the 10‐h infusions, were within one percent of that calculated when the AE were applied to the PB data. Changing the simulated metabolic rate and RQ by the PB “stepping” increased the magnitude of the errors in the second 4.5‐h portion of the test. The variability in errors during the different durations of gas infusions, as well as changing the settings to introduce new simulated metabolic rates, may be due to inaccurate hard calibrations of one or all the individual air mass flow controllers within the PB at certain flow settings.

Propane combustion is the method most used for validation of WRICs (Ravussin et al. 1986; Shechter et al. 2013, 2014, 2015; Arad et al. 2015) and is considered the “gold” standard. Furthermore, instrument grade propane is readily available worldwide (Withers 2001). Moreover, the pressurized lecture bottles, combined with a standard propane welding torch, assure complete combustion (Weast 1985). This is mainly due to its low boiling point (−42°C) which allows instantaneous vaporization (Weast 1985) at the point of ignition. Finally, only a simple needle metering valve, found on all welding torches, is required to regulate the propane gas release from the pressurized lecture bottle. There is a standard configuration for propane specific welding torches that are used for this purpose that allows appropriate air venting in order to maintain the required air‐to‐fuel ratio (Hayword 1988) for complete combustion. Complete oxidation is further assessed by the lack of any kind of odor upon entering the WRIC after a PC test. In the past (Ravussin et al. 1986), 22‐h PC tests were thought to be necessary to simulate a sedentary human subject under similar conditions. However, as institutions tighten fire regulations, they do not allow for unattended PC tests. The finding that only a 10‐h PC test is necessary to validate a WRIC, which is used for 24‐h metabolic studies, is important. This was verified by the fact that a 10‐h PC test produces similar data to that of 22‐h. Moreover, all of the simulated metabolic parameters for this PC test duration were within 1.2 percent of that calculated by Stoichiometry. This shorter duration means that it can be monitored throughout the PC test, alleviating the safety concern of the presence of an unattended open flame within the WRIC. Prior studies have utilized PC tests as short as 6‐h (Ravussin et al. 1986). However, errors in simulated VO_2_ and VCO_2_ were 2–3% (Ravussin et al. 1986) of that of stoichiometry. This was most likely due to incomplete accountability for the sample gas moisture (Ravussin et al. 1986; Melanson et al. 2010; Shechter et al. 2013, 2014, 2015; Arad et al. 2015). Moreover, the burn rate is not linear during the first couple of hours of a PC test (R. Rising, unpublished observations). In ours and the prior study cited (Ravussin et al. 1986) the balance was not connected to the instrumentation for minute‐by‐minute recording of the burn rate. The burn rate was assumed to be linear in the prior (Ravussin et al. 1986) as well as for all three PC test durations evaluated in our study. Therefore, in our study the slightly larger errors in the 6‐h PC tests are most likely due to the assumed initial linear burn rate. The nonlinearity in the burn rate during the first couple of hours of a PC test may be due to the initial rapid reduction in gas pressure in the lecture bottle, along with a slight change in the flame's integrity. After the first couple of hours of the PC test, the pressure reduction in the lecture bottle may be more uniform and the flame more stable. This may result in a more linear burn rate during the rest of the PC test thus reducing the magnitude of errors between stoichiometry and the actual results from the WRIC.

Precision blender infusions have been used in past studies (Charbonnier et al. 1990; Moon et al. 1995; Schoffelen et al. 1997; Tokuyama et al. 2009) to validate WRICs. For example, a new WRIC that was transportable for human studies in the tropics was validated utilizing infusion of a calibration gas containing 20% CO_2_ and 80% nitrogen and also by butane gas combustion. However, in this particular validation (Charbonnier et al. 1990) the additional nitrogen contributed by the PB was accounted for in the simulated metabolic calculations. These investigators achieved simulated metabolic results for the VO_2_ and VCO_2_ that were comparable to that from butane gas combustion (Charbonnier et al. 1990).

Calculations that violate the Haldane transformation (Haldane 1918) during simulated metabolic rates produced by a PB may greatly enhance errors over a short duration infusion test. For example, errors of nearly 6% for each liter of O_2_ occurred when a PB was “stepped” to simulate a human subject exercising for 30 min (Moon et al. 1995). However, errors of <1% in liters per minute of O_2_ were found for a static 24‐h PB infusion test (Moon et al. 1995). It is possible that the calibration coefficients for the blender's mass flow controllers cause more errors when “stepped” to higher flow settings for O_2_ necessary to simulate exercise. In another study, a dual‐respiration WRIC was validated by infusion of only pure CO_2_ by a PB (Schoffelen et al. 1997) for durations of two and 24‐h. However, they still had errors in the concentration of CO_2_ within the WRIC of almost three percent, no matter the test duration. Infusion of only pure CO_2_ in this study greatly reduced the errors associated with violation of the Haldane transformation (Haldane 1918) but, since they utilize membrane dryers (Schoffelen et al. 1997), it is possible that these were due to incomplete removal of sample gas moisture prior to analysis by the O_2_ and CO_2_ sensors (Ravussin et al. 1986; Shechter et al. 2013, 2014, 2015).

The ultimate test of any validation methodology for WRICs is the WM and EB of human subjects that have repeat measurements of 24‐h EE over a long period of time under controlled experimental conditions. Our WRIC was validated by PC prior to two studies (Arad et al. 2015; Hall et al. 2016) where a total of 22 human subjects had repeat measurements of 24‐h EE. In the first study (Arad et al. 2015), Afro‐American women maintained their body weight and EB prior to and after a 14‐day exercise intervention on an outpatient basis. Moreover, their FQ was similar to the RQ obtained from the WRIC. Similar results were found in a second study (Hall et al. 2016) where four men maintained their body weight and EB during the last month on a metabolic ward. However, there was a slightly greater variation in the relationship between the RQs and FQs in this prior study (Hall et al. 2016) versus that found for the Afro‐American women (Arad et al. 2015). The slightly greater differences found between the RQs and FQs for the men residing on the metabolic ward (Hall et al. 2016) may be due to the differences by which their dietary compositions were calculated. In the Hall et al. (2016) study, diet compositions were verified by chemical analysis, whereas in the Afro‐American women they were calculated from a nutrient data base. Furthermore, the formulas utilized to calculate the FQs (Toubro et al. 1998) from the diet compositions of both prior studies (Arad et al. 2015; Hall et al. 2016) were originally derived based on dietary compositions calculated from nutrient data bases. It is possible that utilizing formulas based on nutrient data bases, to calculate FQs for chemically verified dietary compositions, may account for the slight discrepancies found.

In conclusion, we found that a 10‐h PC test provided a more accurate simulation of a sedentary human metabolism compared with that from the PB utilized in our study. Furthermore, this relatively short PC test duration was sufficient to validate WRICs that are used for 24‐h human subject metabolic measurements. This eliminates the nonphysiological aspects of simulated human metabolism produced by the PB. Finally, if a PB is used with the current software for a 10‐h infusion, the AE will reduce the errors due to violation of the Haldane transformation (Haldane 1918) and bring the simulated metabolic values produced close to those obtained by PC.

## Conflict of Interest

Dr. Thomas Foerster is employed by Sable Systems International, who manufactured the respiratory equipment used. However, his contribution solely covers the derivation of the N_2_ corrected respirometer equations, which apply to any PB irrespective of the vendor. He was specifically not involved in calibrations of the instrument, running burn tests, gas infusions, nor data analysis from these tests. All other authors have no financial relationships with any of the vendors mentioned. There was no competing interest among any of the authors of this manuscript.
